# Comparison of Robot-Versus Laparoscopy-Assisted Resection of Choledochal Cysts in Infants Aged Less than 3 Months

**DOI:** 10.3390/jcm15135195

**Published:** 2026-07-02

**Authors:** Ken Chen, Shuhao Zhang, Yuebin Zhang, Duote Cai, Qingjiang Chen, Zhigang Gao

**Affiliations:** Department of General Surgery, Children’s Hospital, Zhejiang University School of Medicine, National Clinical Research Center for Children and Adolescent’s Health and Diseases, Hangzhou 310052, China; pwck@zju.edu.cn (K.C.); 6519040@zju.edu.cn (S.Z.); pwzyb@zju.edu.cn (Y.Z.); cdt1240@zju.edu.cn (D.C.); chenqj0157@zju.edu.cn (Q.C.)

**Keywords:** choledochal cysts, young infants, robotic surgery, laparoscopic surgery

## Abstract

**Background:** The utilization of robot-assisted surgery in pediatric patients is increasing, with particularly notable advantages in complex reconstructive procedures. This study aims to evaluate the safety and efficacy of robotic-assisted resection of choledochal cysts in infants aged less than 3 months. **Methods:** A total of 73 infants with choledochal cysts who were admitted to the Department of General Surgery, Children’s Hospital of Zhejiang University School of Medicine, between April 2019 and December 2025 were included. The patients were divided into a robotic-assisted surgery (RAS) group (*n* = 39) and a laparoscopic-assisted surgery (LAS) group (*n* = 34). Clinical data, including demographic information, laboratory indexes, surgical data, and prognostic data, were retrospectively reviewed, and the Mann–Whitney U test, independent-samples *t*-test, and Fisher’s exact test were used for statistical analysis. **Results:** The groups were comparable in terms of age, sex, weight, pre- and postoperative biochemical markers, fasting time, cyst diameter, and operative time. Overall, 80.8% of cases were prenatally detected. The RAS group had a significantly shorter postoperative hospital stay (*p* = 0.004, Z = −2.864), drainage tube duration (*p* = 0.002, Z = −3.100), and hepaticojejunostomy time (*p* < 0.0001, df = 71, 95%CI (−5.70, −3.04)) compared to the LAS group. In the LAS group, three patients developed anastomotic fistulas, all of whom required reoperation, and one patient developed adhesive bowel obstruction, whereas in the RAS group, one patient developed incision infection, one developed cholangitis, one developed adhesive bowel obstruction, and one presented with postoperative liver function abnormalities. The hospitalization cost in the LAS group was significantly lower than that in the RAS group (*p* < 0.0001, Z = −5.468). **Conclusions:** In experienced pediatric centers, robotic-assisted resection of choledochal cysts is safe and effective for infants aged less than 3 months and deserves further exploration.

## 1. Introduction

Congenital biliary dilation, also known as choledochal cyst (CC), primarily affects the intra- or extrahepatic bile ducts. Its incidence in Asian countries ranges from 1 in 1000 to 1 in 3000, significantly higher than that observed in European and American countries, which ranges from 1 in 150,000 to 1 in 50,000. The male-to-female ratio is approximately 1:3–1:4 [1,2]. The clinical manifestations of a CC differ based on age; neonates and young infants may present with obstructive jaundice, acholic stools, abdominal pain, or even evidence of advanced liver fibrosis [3]. Laparoscopic-assisted surgery is the preferred surgical approach for treating CC. With advancements in the robotic surgical system, particularly its adaptation for pediatric use, robotic-assisted surgery (RAS) offers distinct advantages over traditional laparoscopy, especially in terms of biliary anatomy visualization and hepaticojejunostomy [4]. Therefore, many pediatric medical centers have started to perform robotic-assisted resection of choledochal cysts (RACCs), supported by large-sample studies confirming the safety and efficacy of the robotic system. However, the application of robotic systems in young infants, particularly those under three months of age or weighing less than 10 kg, requires additional clinical cases and long-term follow-up data to substantiate its feasibility [5,6]. The current literature on RACC in this age group is limited to a few case series. This study provides additional supportive data and aims to compare robotic surgery with laparoscopic surgery for CC treatment, specifically evaluating the safety and efficacy of the robotic approach in patients under 3 months of age.

## 2. Materials and Methods

### 2.1. Clinical Characteristics and Study Design

The retrospective study cohort comprised 73 CC patients (16 males and 57 females) at the Department of General Surgery of the Children’s Hospital of Zhejiang University School of Medicine between April 2019 and December 2025. The surgical approach was chosen voluntarily by the parents. Based on our clinical experience, the primary factors influencing decision making are the family’s economic level and the guardians’ level of awareness of robotic surgery. Then, patients were categorized into two groups: laparoscopic- (LAS, *n* = 34) and robotic-assisted surgery (RAS, *n* = 39). The robotic surgery system is Da Vinci Xi (Intuitive Surgical, Inc., Sunnyvale, CA, USA). All procedures were performed by the same surgical team. Among the RAS group, 9 patients were male, and 30 were female, and in the LAS group, there were 7 males and 27 females. Demographic information, including sex, age, and weight, showed no significant differences between the two groups (*p* > 0.05). Clinical data, including preoperative symptoms, Todani classification, biochemical indexes, cystic diameter, operative time, hospital stay duration, abdominal drainage tube duration, costs, fasting times, and postoperative complications, are presented in Table 1, Table 2 and Table 3.

### 2.2. Inclusion and Exclusion Criteria

The inclusion criteria were as follows: (1) children <3 months of age; (2) the Children’s Hospital of Zhejiang University School of Medicine is the first hospital that patients visited; (3) patients do not have a history of any other abdominal surgeries; and 4) absence of co-existing diseases.

The exclusion criteria were as follows: (1) children >3 months of age; (2) the Children’s Hospital of Zhejiang University School of Medicine was not the first hospital that patients visited; (3) patients had a history of other abdominal surgeries; and (4) co-existence of other congenital diseases.

### 2.3. Operative Procedure and Docking Position

**Figure 1:**
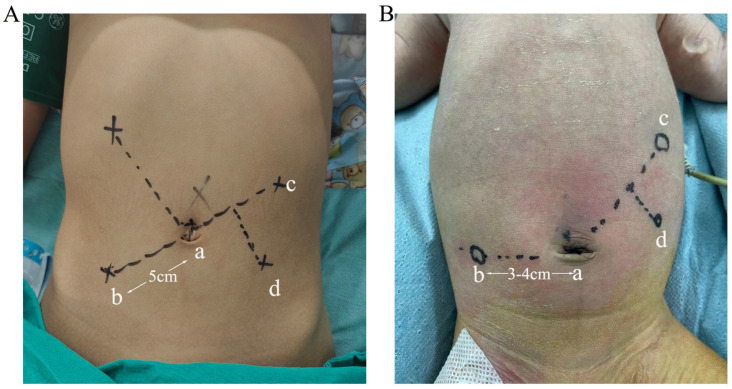
Docking position for robotic surgery in patients with choledochal cyst. (**A**,**B**) Docking positions for patients >3 months and < 3 months of age, respectively: an 8 mm camera port (port a), two 8 mm working ports (ports b and c), and a 5 mm assistant port (d).

Robotic surgery: (1) After anesthesia, patients are positioned in the supine position, and the pneumoperitoneum is established through the umbilicus (pneumoperitoneal pressure: 6–8 mmHg). Four ports are created (the locations of four ports of children >3 and <3 months are shown in Figure 1A and Figure 1B, respectively), including an 8 mm port passing through the umbilicus to be used as a camera port (port a), two 8 mm working ports (ports b and c), and a 5 mm assistant port (port d). (2) The jejunum is secured 20 cm distal to the ligament of Treitz. After laparoscope withdrawal and pneumoperitoneum evacuation, the incision is extended to exteriorize the loop. Mesenteric vessels are ligated, and the bowel is transected with a stapler. The distal biliary limb is closed with 4–0 absorbable sutures (running full-thickness plus interrupted seromuscular). An end-to-side anastomosis is made between the proximal jejunum and a segment 25 cm downstream from the closed end, with an anti-reflux valve. The bowel is returned, and the pneumoperitoneum is restored. (3) Then, patients are positioned in the reverse Trendelenburg position, followed by installation of robotic arms. The gallbladder and ligamentum teres are suspended. The cystic triangle is dissected, the cystic artery is divided, and the dissection is carried to the cystic duct insertion. The cyst wall is dissected distally with pancreatic protection, and the distal stump is clipped with Hem-o-lok. The proximal choledochal cyst is dissected to about 1 cm below the hepatic duct confluence. (4) An avascular window is created in the mesentery left to the middle colic artery, and the biliary limb is passed to the retromesocolic plane. The bowel wall is fixed to the mesentery with two interrupted 5–0 sutures. A longitudinal incision is made on the antimesenteric border 1 cm proximal to the blind end, and an end-to-side hepato-biliary anastomosis is completed with continuous full-thickness 5–0 sutures.

Laparoscopic surgery: After anesthesia, patients are positioned in the supine position, and the pneumoperitoneum is established through the umbilicus (pneumoperitoneal pressure: 6–8 mmHg). Four ports are created, including a 5 mm port passing through the umbilicus to be used as a camera port, two 5 mm working ports (the midclavicular line at the umbilical level of the right midabdomen and the surface projection of the gallbladder in the right upper quadrant), and a 5 mm assistant port (approximately 2 cm below the costal margin along the midclavicular line of the left upper quadrant). The following steps are the same as those of robotic surgery.

### 2.4. Evaluations of Study Outcomes

The primary outcomes included (1) incidence of postoperative complications; (2) conversion to laparotomy; (3) rate of reoperation; and (4) time of hepaticojejunostomy.

The secondary outcomes included (1) length of postoperative stay; (2) postoperative fasting time; and (3) removal of the abdominal drainage tube.

### 2.5. Statistical Analysis

Statistical analyses were performed using SPSS 22.0 and GraphPad Prism 6. Normality of continuous data was assessed with the Kolmogorov–Smirnov test. Non-normally distributed data are expressed as the median (interquartile range) and were compared using the Mann–Whitney U test; normally distributed data are reported as the mean ± standard deviation and were compared using the independent-samples *t*-test. Categorical variables were analyzed with Fisher’s exact test. All tests were two-tailed, and a *p*-value < 0.05 was considered statistically significant.

## 3. Results

### 3.1. Clinical Characteristics for Patients in LAS and RAS Groups

The LAS group consisted of 7 male and 27 female patients, with a median age of 54.00 days (interquartile range [IQR], 37.25–63.25 days) and a mean weight of 4.93 ± 1.05 kg; the RAS group consisted of 9 male and 30 female patients, with a mean age of 64.92 ± 32.18 days and a mean weight of 5.17 ± 1.43 kg. In the LAS group, 13 patients presented with preoperative jaundice and 13 had preoperative abnormal liver function; in the RAS group, 19 patients had preoperative jaundice, and 8 had preoperative abnormal liver function. Regarding Todani classification, the LAS group comprised 22 patients with type I and 12 with type IV, and 26 were prenatally diagnosed; the RAS group comprised 28 patients with type I, 1 with type II, and 10 with type IV, and 33 were prenatally diagnosed (Table 1). The median cystic diameter in the LAS group was 32.50 mm (IQR, 23.98–42.75 mm), 12 of whom had complications with intrahepatic bile duct dilations; that in the RAS group was 26.00 mm (IQR, 21.56–37.00 mm), 10 of whom had complications with intrahepatic bile duct dilations. The median hospitalization cost in the LAS group was significantly lower than that in the RAS group [30,058.59 RMB (IQR, 26,302.30–40,640.33 RMB) vs. 76,635.08 RMB (IQR, 74,209.26–82,271.50 RMB), *p* < 0.0001, Z = −5.468] (Table 1).

### 3.2. Intra- and Postoperative Data for Patients in RAS and LAS Groups

**Figure 2:**
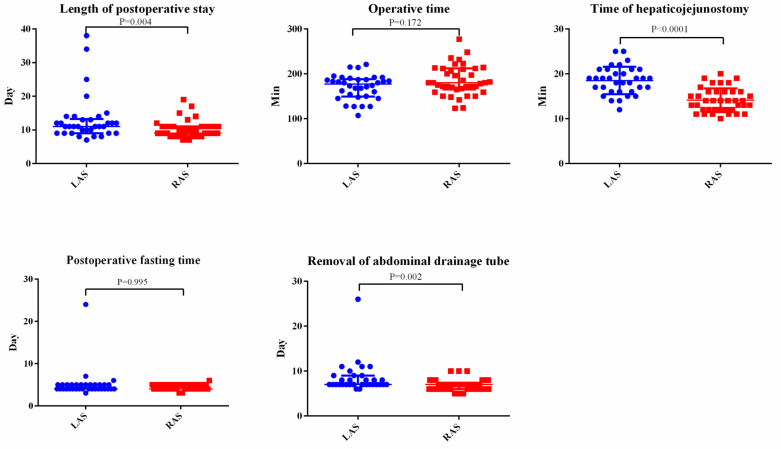
Comparisons of treatment outcomes between the LAS and RAS groups.

No conversion to open laparotomy was observed in either group. The mean operative time of the LAS group was 170.50 ± 27.31 min, and that of the RAS group was 180.00 min (IQR, 165.00–212.00 min), with no statistically significant difference between the two groups (*p* = 0.172, Z = –1.366). However, the time of hepaticojejunostomy in the LAS group was significantly longer than that in the RAS group [18.50 ± 3.06 min vs. 14.13 ± 2.63, *p* < 0.0001, df = 71, 95%CI (−5.70, −3.04)]. The length of postoperative stay in the LAS group was also significantly longer than that in the RAS group [11.00 days (IQR, 9.00–13.25 days) vs. 9.0 days (IQR, 8.0–11.0 days), *p* = 0.004, Z = −2.864]. Postoperative fasting time showed no significant differences between the two groups (*p* = 0.959), while the abdominal drainage tube was removed earlier in the RAS group than in the LAS group (*p* = 0.002, Z = −3.100) (Table 3 and Figure 2). The median duration of follow-up in the LAS group was 1943.00 days (IQR, 702.50–2457.00 days), while that in the RAS group was 846.77 ± 419.01 days.

### 3.3. Postoperative Complications in LAS and RAS Groups

In the LAS group, three patients underwent reoperation due to anastomotic fistulas on postoperative days 6, 8, and 21; their ages were 38, 87, and 18 days, and their weights were 5.0, 6.2, and 3.9 kg, respectively. One patient developed adhesive bowel obstruction 6 months postoperatively and received conservative management. In the RAS group, one patient developed an incision infection and received incision and drainage of the abscess. In addition, one patient developed cholangitis 2 months postoperatively, one patient developed adhesive bowel obstruction 2 years postoperatively, and one patient developed cholangitis 3 weeks postoperatively; these patients recovered after conservative management. Between these two groups, the postoperative complications showed no significant difference (*p* = 1.000).

## 4. Discussion

Approximately 80% of CBD cases are diagnosed during childhood and within the first decade of life [7]. With advancements in ultrasound technology, there has been a notable increase in the prenatal and early postnatal diagnosis of CC [8,9,10]. In this study, the proportion of patients under three months of age diagnosed prenatally reached as high as 80.8%. Prenatal examinations have revealed a trend of decreasing age for CC occurrence, consequently leading to progressively younger ages at surgical intervention [11,12]. With the recent widespread adoption of robotic surgery in pediatric surgery, RACC has gained increasing favor among surgeons. Accordingly, the present study aims to evaluate the safety and efficacy of RACC in infants aged less than 3 months.

### 4.1. Timing of Surgery for CC

**Figure 3:**
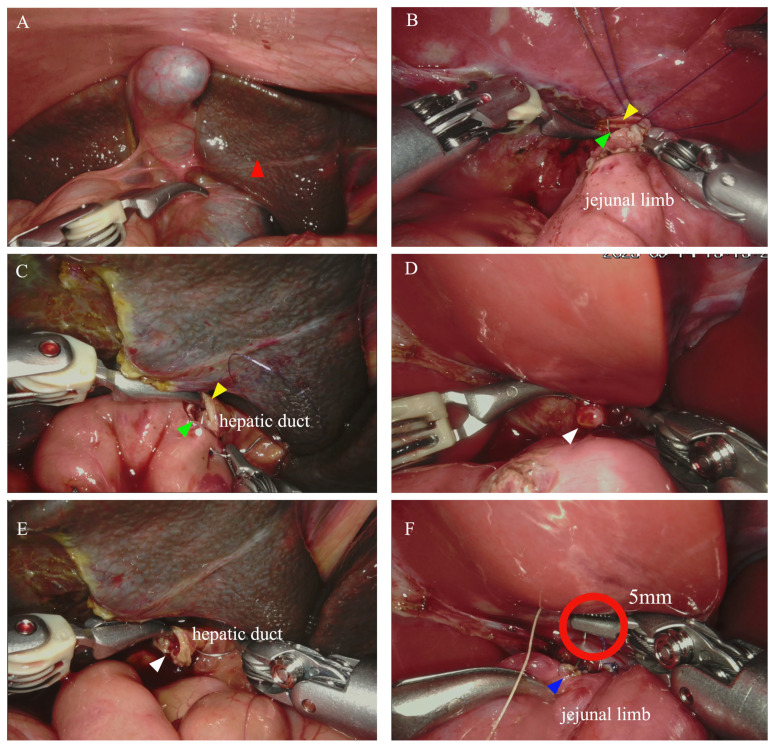
Intraoperative pictures. (**A**) Signs of liver cirrhosis (red arrow) were observed in one neonatal patient. (**B**,**C**) The surgical view of the anterior (yellow arrow) and posterior (green arrow) walls of the common bile duct was clear. (**D**,**E**) The small hepatic duct (white arrow) in neonates was clearly visible under the magnified view of the robotic system, even though its diameter was approximately 2–3 mm. Under this view, the length of the robotic instrument’s tip within the red circle is approximately 5 mm. (**F**) During the procedure, precise repair was performed on the anastomosis even the diameter is less than 5 mm (the red circle marks the tip of the needle holder, with an approximate length of 5 mm), which was not securely sutured.

According to the Japanese clinical guidelines for pancreaticobiliary maljunction, symptomatic CC should be surgically addressed in the presence of symptoms, while elective surgery is recommended for asymptomatic CC at approximately 3–6 months of age to prevent anastomotic complications [13]. However, some studies advocate for immediate surgical intervention upon diagnosis, as prenatally diagnosed patients often exhibit a high rate of symptoms and complications requiring careful follow-up and management after birth [14,15]. In our study, the proportions of patients with preoperative liver function abnormalities or cholestasis in the LAS and RAS groups were as high as 55.9% and 53.8%, respectively. Notably, one neonatal patient even demonstrated a tendency towards liver cirrhosis (Figure 3A). Currently, there is no consensus on the indications or appropriate timing for laparoscopic surgery in infants [16,17]. Based on our center’s experience, we tend to favor early aggressive surgical intervention when the cystic diameter exceeds 30 mm, the cyst manifests high tension, or patients suffer from biliary obstruction symptoms (e.g., jaundice, clay-colored stools). This principle aligns with previous findings indicating that a cystic diameter over 30 mm or the presence of intrahepatic bile duct dilation warrants early surgical intervention [18]. 

### 4.2. Modified Strategies for Robotic Surgery in Infants <3 Months

The limited intraperitoneal space of neonates or small infants posed the greatest challenge for minimally invasive surgery [19,20]. In some centers, the conversion rate of laparoscopic procedures in young children even reached up to 30% [21]. As for robotic surgery, the conflict between the larger surgical instruments and the limited abdominal space in young children has been further magnified. Previous research has indicated that RAS was primarily suitable for patients older than 2 years and weighing over 15 kg [22]. In 2010, Dawrant [23] et al. reported successful RAS in five children with CC weighing less than 10 kg and were the first to affirm the advantages of RAS in young children. In 2024, Ishii [24] further confirmed the safety of RAS in patients weighing less than 10 kg by expanding the sample size. However, both of these studies lacked follow-up data on long-term complications. Subsequently, Xie [25] and Rong [26] focused on the application of RAS on infants under 1 year of age, and all procedures were successfully completed without complications. Liao [27] even explored the application of RAS in neonates; nevertheless, this study also lacked long-term follow-up data (Table 4). In the present study, we successfully completed 39 RASs in young infants with CC, 6 of whom were neonates; all procedures were successfully performed without conversion to laparotomy, confirming that RAS is applicable to infants under 3 months of age. Key measures that facilitated this achievement included the following: (1) Trocar distance: Although the recommended distance for trocar placement in RAS was 8–10 cm, we were able to reduce this limit to 3–4 cm according to our clinical practices without experiencing instrument collisions during operations. Additionally, the trocars on the right side of the abdomen were positioned as close to the mid-axillary line as possible to increase the distance between the robotic arms. (2) Trocar placement: The trocar placements on the left and right abdominal sides should be as far away as possible from the gallbladder, and the trocar on the right abdominal side should also avoid being positioned too low to prevent injury to the iliac vessels and iliac bones. Therefore, the trocar placement for neonates did not strictly adhere to the triangular arrangement (the lateral ports align vertically with the umbilicus and the surgical site, Figure 1A). Instead, we opted for a parallel placement near the umbilicus and as close to the mid-axillary line as possible (Figure 1B). Additionally, we will use rubber covers on the trocar in the future to increase the friction between the metal trocar and the skin, creating extra abdominal cavity space by retracting the abdominal skin. (3) Patients’ position: Based on the dorsal elevated position, we further elevated the child’s body using silicone pads to increase the working space for the robotic arms.

### 4.3. Safety and Efficacy of Robotic Surgery in Infants <3 Months

Safety and efficacy are crucial when evaluating the suitability of robotic surgery for infants under three months. In this study, three children in the LAS group required reoperation due to postoperative anastomotic leak, and the average hospital stay for these three patients was 32.3 days. No patient in the RAS group required reoperation. The advantages of the robotic system—such as 3D visualization, a 7-degree range of motion, and tremor elimination—enhanced dexterity when handling the delicate common bile duct of neonates, and the surgical field of view was clearer during deep anastomosis (Figure 3B,C). Benefiting from these advantages, our attending surgeon demonstrated markedly greater confidence during hepaticojejunostomy, especially for neonates with a common bile duct diameter less than 5 mm (Figure 3D,E). We even identified a case of anastomotic leak in the RAS group during the procedure and performed a precise repair (Figure 3F). Furthermore, with respect to hepaticojejunostomy, the time was shorter in the RAS group; in terms of hospital stay, the RAS group had less wound exudation, allowing earlier removal of the abdominal drainage tube and earlier discharge. Regarding long-term complications, our mean follow-up duration was 846.77 ± 419.01 days, which far exceeds the peak incidence period for anastomotic stricture at 233.5 days [28]. During this period, no Clavien–Dindo grade > II complications were observed. Therefore, the advantages of robotic surgery are most prominently reflected in anastomosis management, management of complex accessory hepatic duct variations, and more reliable distal ligation of the cyst. Furthermore, for surgeons with extensive laparoscopic experience, the learning curve for RAS is only 17 cases [29], which is another important advantage of RAS for complex reconstructive procedures in children. Based on our single-center preliminary experience, RACC represents a safe and effective surgical technique; however, its long-term outcomes remain to be further validated. Also, it is undeniable that the high cost of RAS is one of the most important factors limiting its widespread adoption.

This study has several limitations. First, this is a single-center study with a relatively small sample size, which may be underpowered in detecting subtle yet clinically meaningful differences in rare complications and may elevate the risk of Type II error. Second, it was a retrospective study. Although surgical approaches were voluntarily chosen by patients, selection bias may still exist. Third, as a pediatric medical center with extensive experience in the management of CC (with over 1000 cases), our techniques may not be generalizable to all institutions. In future studies, we plan to expand the sample size and conduct multicenter studies in collaboration with other pediatric centers. Additionally, the follow-up duration of the RAS group was significantly shorter compared with that of the LAS group; therefore, we will further prolong the follow-up period for infants under three months of age in subsequent research to further evaluate the safety and efficacy of RACC in young infants.

## 5. Conclusions

In summary, early surgical intervention for CC is not followed by a high risk of complications and may prevent the onset of preoperative complications, offering excellent outcomes, particularly with minimally invasive techniques. In pediatric centers with extensive experience in CC management, RACC is safe and effective for young infants aged less than 3 months and deserves further exploration.

## Figures and Tables

**Table 1:** Clinical characteristics of patients in LAS and RAS groups.

	LAS (*n* = 34)	RAS (*n* = 39)	*p* Value
Sex (male:female)	7:27	9:30	*p* = 1.000
Age (days)	54.00 (37.25–63.25)	64.92 ± 32.18	*p* = 0.179, Z = −1.344
Weight (kg)	4.93 ± 1.05	5.17 ± 1.43	*p* = 0.412, df = 71, 95%CI (−0.35, 0.83)
Preoperative symptom			
Jaundice	13	19	*p* = 1.000
Liver function abnormalities	13	8	*p* = 1.000
Anatomical classification			
Todani I	22	28	/
Todani II	0	1	/
Todani III	0	0	/
Todani IV	12	10	/
Patients with prenatal discovery (*n*)	26	33	*p* = 1.000
Cost (RMB)	30,058.59 (26,302.30–40,640.33)	76,635.08 (74,209.26–82,271.50)	*p* < 0.0001, Z = −5.468

LAS: laparoscopy-assisted surgery; RAS: robot-assisted surgery.

**Table 2:** Preoperative and postoperative examinations of patients in LAS and RAS groups.

	LAS (*n* = 34)	RAS (*n* = 39)	*p* Value	Z Statistic
Initial biochemical indexes				
GGT (U/L)	144.50 (56.75–410.75)	139.00 (39.00–462.00)	0.439	−0.774
ALT (U/L)	41.50 (23.00–85.00)	36.00 (19.00–66.00)	0.239	−1.178
AST (U/L)	61.00 (38.75–130.00)	61.00 (36.00–94.00)	0.580	−0.553
TBIL (μmol/L)	34.55 (13.33–81.80)	23.30 (7.50–85.20)	0.410	−0.824
DBIL (μmol/L)	7.80 (3.50–29.63)	6.90 (2.10–25.10)	0.455	−0.747
IBIL (μmol/L)	22.80 (7.98–44.98)	10.90 (5.80–38.10)	0.366	−0.962
AMY (U/L)	12.85 (8.35–46.80)	14.50 (7.00–48.60)	0.991	−0.011
Final biochemical indexes				
GGT (U/L)	59.50 (28.50–168.25)	37.00 (16.00–97.00)	0.054	−1.930
ALT (U/L)	27.50 (16.00–41.75)	19.00 (13.00–28.00)	0.075	−1.782
AST (U/L)	43.00 (37.00–67.00)	35.00 (30.00–50.00)	0.018	−2.368
TBIL (μmol/L)	10.10 (5.95–20.40)	9.00 (5.50–21.20)	0.674	−0.420
DBIL (μmol/L)	2.85 (1.70–4.60)	3.10 (1.60–5.70)	0.851	−0.188
IBIL (μmol/L)	8.00 (4.075–13.80)	5.80 (3.50–16.30)	0.686	−0.404
AMY (U/L)	14.35 (8.60–35.98)	23.00 (12.40–58.00)	0.060	−1.880
Cystic diameter (mm)	32.50 (23.98–42.75)	26.00 (21.56–37.00)	0.232	−1.195
Number of intrahepatic bile duct dilations (*n*)	12	10	*p* = 1.000	

GGT: Gamma-glutamyl transpeptidase; ALT: alanine transaminase; AST: aspartate transaminase; TBIL: total bilirubin; DBIL: direct bilirubin; IBIL: indirect bilirubin; AMY: amylase. Normal range: GGT (9–150 U/L), ALT (8–71 U/L), AST (21–80 U/L), TBIL (5–21 μmol/L), DBIL (0–5.1 μmol/L), IBIL (1–20 μmol/L), AMY (40–132 μmol/L).

**Table 3:** Treatment outcomes for patients in LAS and RAS groups.

	LAS (*n* = 34)	RAS (*n* = 39)	*p* Value
Length of postoperative stay (days)	11.00 (9.00–13.25)	9.0 (8.0–11.0)	*p* = 0.004, Z = −2.864
Operative time (min)	170.50 ± 27.31	180.00 (165.00–212.00)	*p* = 0.172, Z = −1.366
Time of hepaticojejunostomy (min)	18.50 ± 3.06	14.13 ± 2.63	*p* < 0.0001, df = 71, 95%CI (−5.70, −3.04)
Postoperative fasting time (days)	4.00 (4.00–5.00)	4.00 (4.00–5.00)	*p* = 0.995, Z = −0.006
Removal of abdominal drainage tube (days)	7.00 (7.00–9.00)	7.0 (6.0–7.0)	*p* = 0.002, Z = −3.100
Postoperative complications (total)	4	4	*p* = 1.000
Anastomotic fistula (*n*)	3	0	/
Incision infection (*n*)	0	1	/
Anastomotic stricture (*n*)	0	0	/
Cholangitis (*n*)	0	1	/
Adhesive bowel obstruction (*n*)	1	1	/
Liver function abnormalities	0	1	/
Conversion to laparotomy	0	0	/
Reoperation (*n*)	3	0	/
Duration of follow-up (days)	1943.00 (702.50–2457.00)	846.77 ± 419.01	*p* = 0.007, Z = −2.716

Operative time: the duration from skin incision to abdominal closure. Time of hepaticojejunostomy: the first suture to the final suture for hepaticojejunostomy.

**Table 4:** Comparative studies on robotic surgery in recent years.

Author	Year	Number of Patients	Age	Operative Time	Follow-up Time	Complications	Conversion to Laparotomy	Conclusion
Liao [27]	2025	32	21.17 ± 6.56 days	208.54 ± 10.43 min	3 months	0	0	Da Vinci robotic-assisted surgery is safe and feasible for the treatment of neonatal CCC.
Ishii [24]	2024	10	186 days (IQR: 18–741)	345 min (IQR: 297–438)	/	Anastomotic fistula (*n* = 1)	0	RAS can be safely and precisely implemented for infants weighing ≤ 10 kg.
Rong [26]	2022	28	4.9 months (IQR: 3.1–9.1)	204 min (IQR: 185–225)	3.0 ± 1.6 years	0	0	Robot-assisted cyst resection and hepaticojejunostomy are feasible and safe in infants≤ 1 year old.
Xie [25]	2021	10	8.50 months (IQR: 7.75–10.25)	219.50 (11.55) min	24 months	Adhesive bowel obstruction (*n* = 1)	0	The Da Vinci surgical system is a safe and feasible form of treatment for choledochal cysts in children <1 year old.
Dawrant [23]	2010	5	1 year (IQR: 0.5–1.4)	482 ± 55 min	/	0	0	The technique is safe and effective in children weighing less than 10 kg.

## Data Availability

The data presented in this study are available upon request from the corresponding author due to ethical reasons.
